# Supplementary material for: Rosemary essential oil and its components 1,8-cineole and α-pinene induce ROS-dependent lethality and ROS-independent virulence inhibition in Candida albicans
Source: PLoS One. 2022 Nov 16;17(11):e0277097. doi: 10.1371/journal.pone.0277097 (PMC9668159; doi:10.1371/journal.pone.0277097)
Supplement: S1 Fig — (DOCX) [file pone.0277097.s001.docx]

**
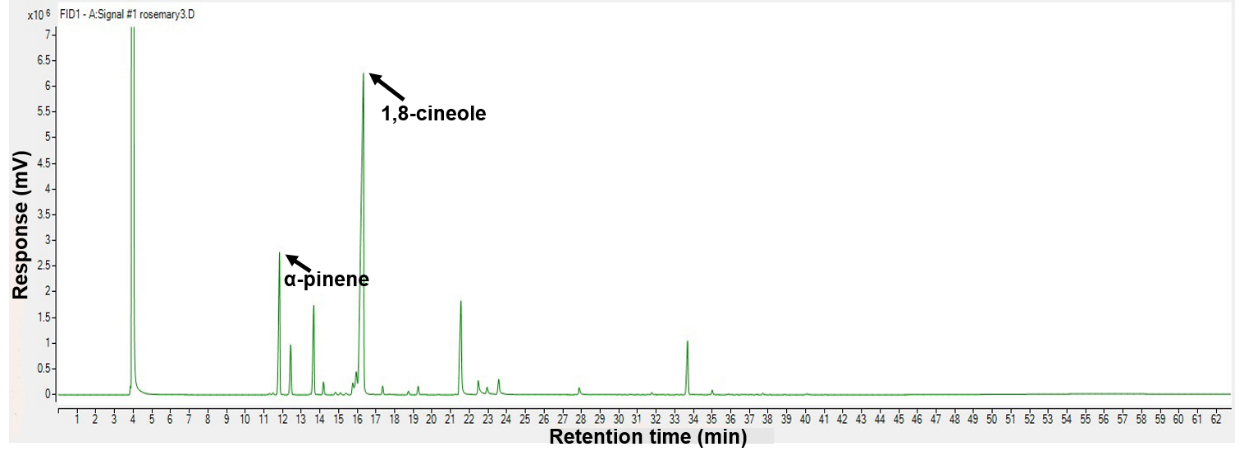
**

**S1** **Fig. Gas chromatogram of RM oil**.

Peak areas show the major component to be 1,8-cineole, followed by α-pinene.
