# Supplementary material for: Rosemary essential oil and its components 1,8-cineole and α-pinene induce ROS-dependent lethality and ROS-independent virulence inhibition in Candida albicans
Source: PLoS One. 2022 Nov 16;17(11):e0277097. doi: 10.1371/journal.pone.0277097 (PMC9668159; doi:10.1371/journal.pone.0277097)
Supplement: S2 Fig — (DOCX) [file pone.0277097.s002.docx]

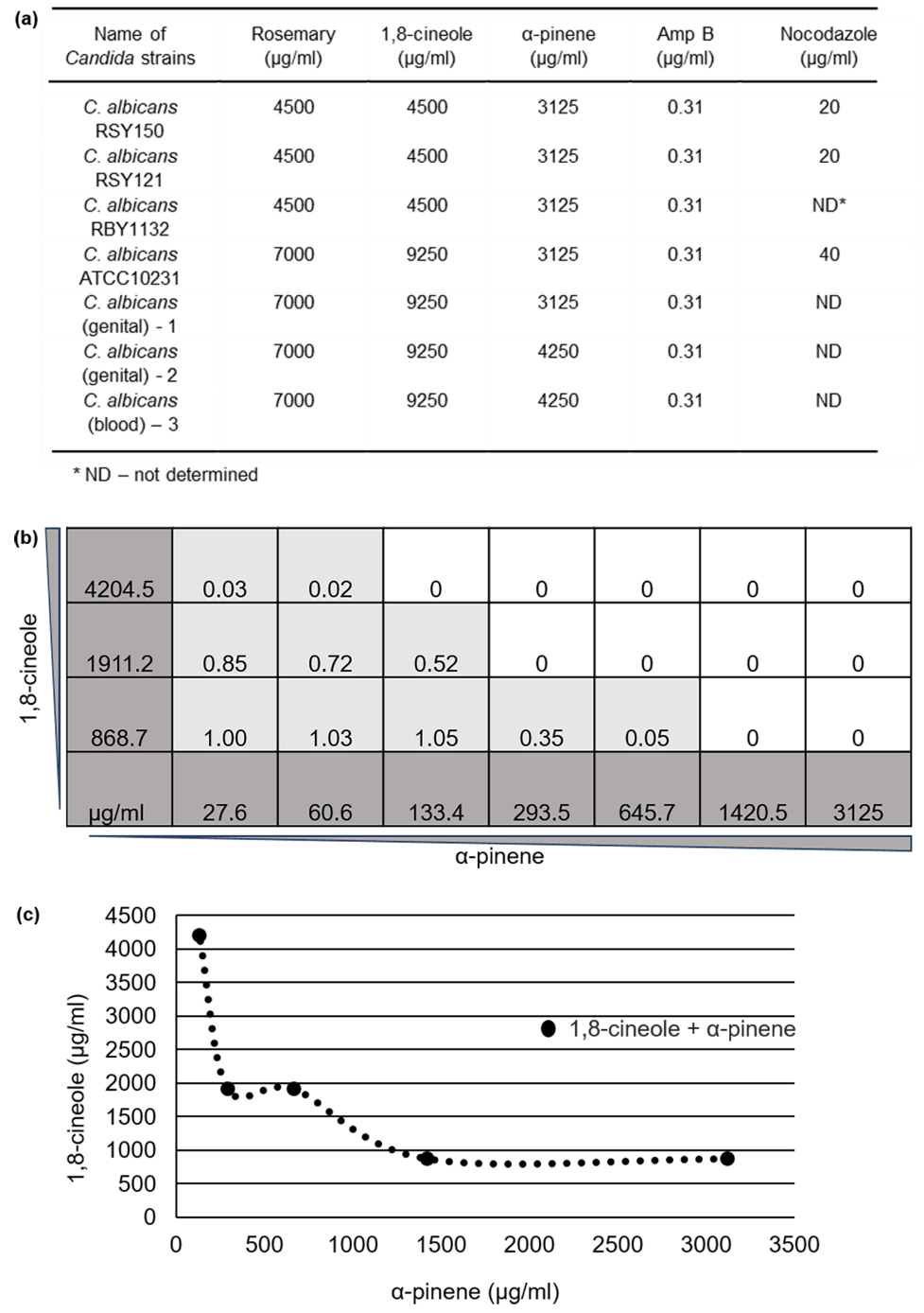


**S2 Fig. MIC of various *C. albicans*** **strains and EOC synergy.**

(a) MIC values of different *C. albicans* strains exposed to rosemary oil and its components 1,8-cineole and α-pinene from three biological replicates, for which the typical error for a null OD_600_ reading was 0.01. (b) The checkerboard assay revealed a 2-fold (4205 to 1911 μg/ml) and 10-fold (3125 to 293 μg/ml) reduction in the MIC for 1,8-cineole and α-pinene, respectively. Dark grey is oil concentration, light grey shows visible growth and white is no growth (OD_600_). (c)The isobologram derived from the checkerboard assay visually shows partial synergy.
