# Supplementary material for: Rosemary essential oil and its components 1,8-cineole and α-pinene induce ROS-dependent lethality and ROS-independent virulence inhibition in Candida albicans
Source: PLoS One. 2022 Nov 16;17(11):e0277097. doi: 10.1371/journal.pone.0277097 (PMC9668159; doi:10.1371/journal.pone.0277097)
Supplement: S3 Fig — (DOCX) [file pone.0277097.s003.docx]

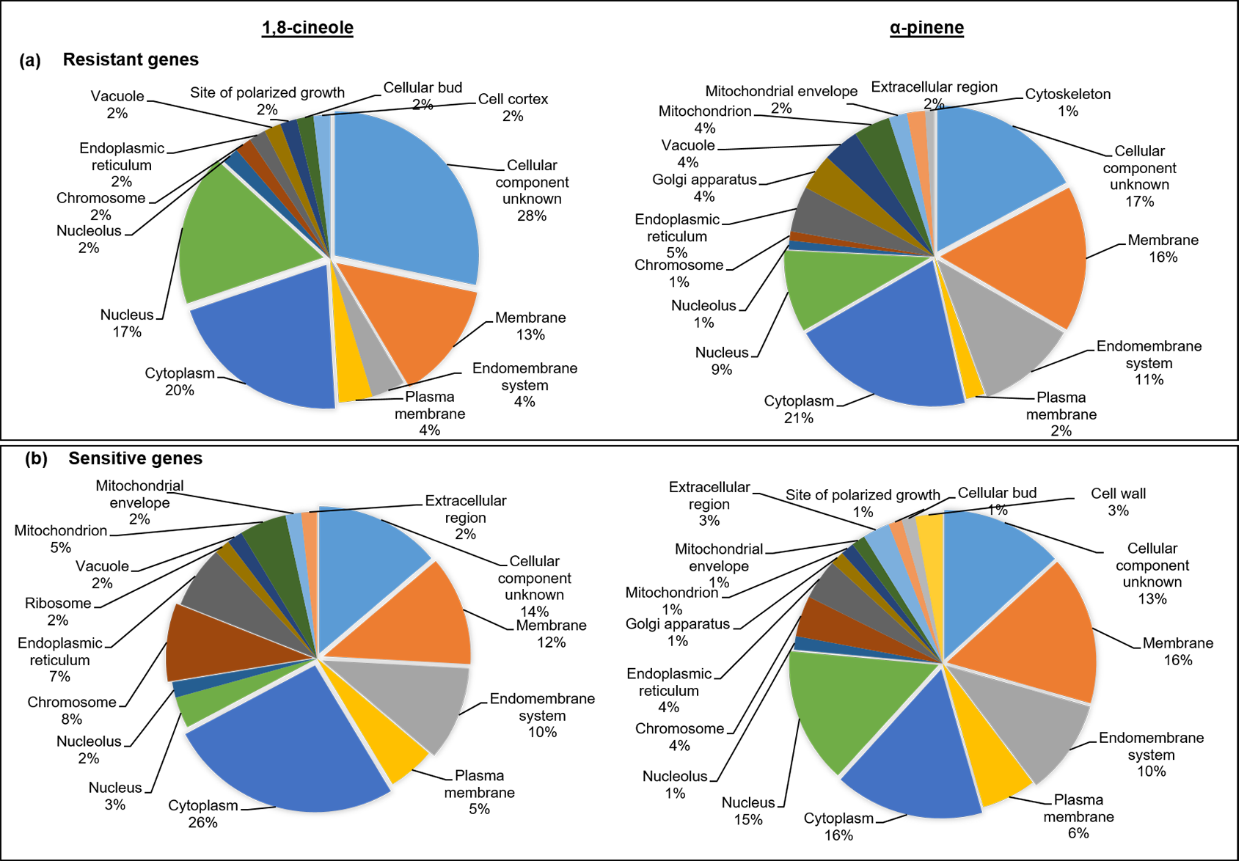


**S3 Fig. *C. albicans* genes conferring resistance and sensitivity to EOCs.**

Cluster frequency outcome constructed using the CGD GO-Slim component mapper indicates that the plasma membrane and several membranous organelles are affected to a greater degree than the other organelles.
