# Supplementary material for: Rosemary essential oil and its components 1,8-cineole and α-pinene induce ROS-dependent lethality and ROS-independent virulence inhibition in Candida albicans
Source: PLoS One. 2022 Nov 16;17(11):e0277097. doi: 10.1371/journal.pone.0277097 (PMC9668159; doi:10.1371/journal.pone.0277097)
Supplement: S4 Fig — (DOCX) [file pone.0277097.s004.docx]

**
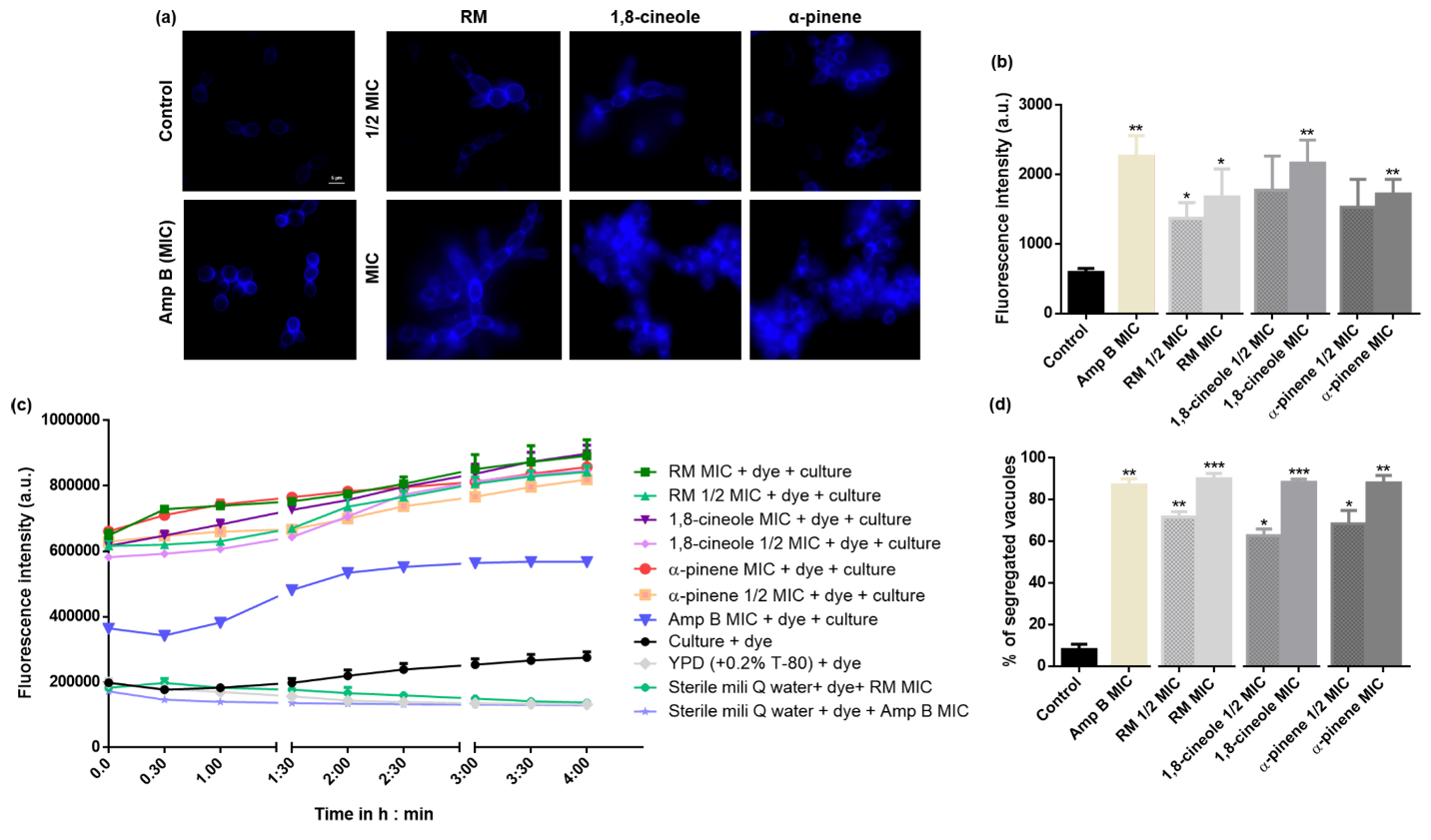
**

**S4 Fig. Effects of RM oil and its major components 1,8-cineole and α-pinene on *C.***

***albicans* ATCC10231.**

(a,b) EO(C) exposure induces cell separation defects and influences increased chitin production in ATCC10231. Scale bars for control are 5 μm and applicable for all. (b) Membrane potential assay of ATCC10231 shows a significant increase (*p* < 0.05) in fluorescence intensity of dye Dis-C2(3), over the course of a 4 h exposure to the oils at 1/2 MIC and MIC. (d) Impact on vacuoles after 4 h treatment with RM oil and its components 1,8-cineole and α-pinene. (b,c) Data are presented as the mean ± SEM of three biological replicates, with 300 cells per replicate, for which statistical significance (***, *p* < 0.001; **, *p* < 0.01; *, *p* < 0.05) as analysed by an unpaired Student’s *t*-test.
