# Supplementary material for: Rosemary essential oil and its components 1,8-cineole and α-pinene induce ROS-dependent lethality and ROS-independent virulence inhibition in Candida albicans
Source: PLoS One. 2022 Nov 16;17(11):e0277097. doi: 10.1371/journal.pone.0277097 (PMC9668159; doi:10.1371/journal.pone.0277097)
Supplement: S5 Fig — (DOCX) [file pone.0277097.s005.docx]

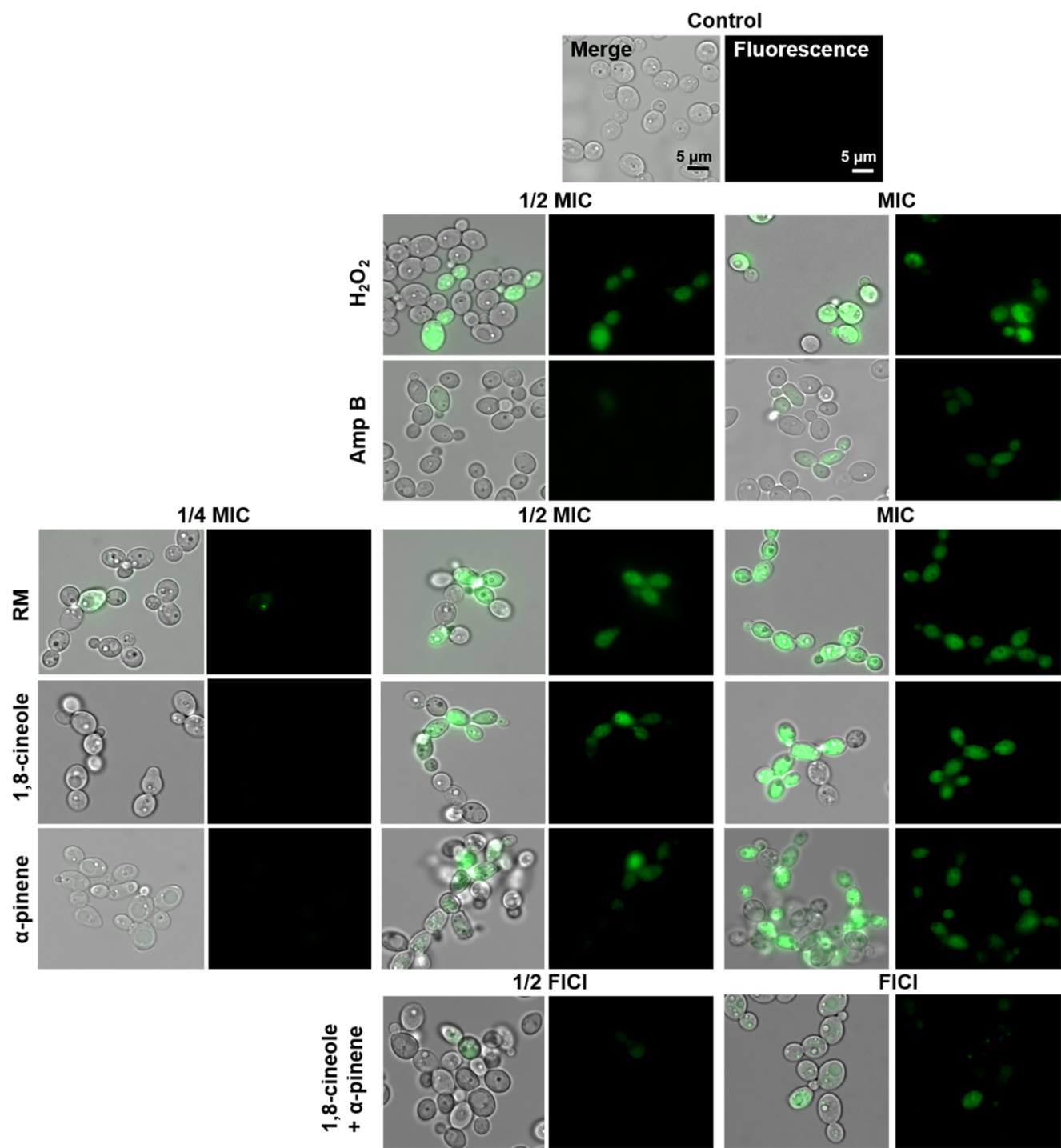


**S5 Fig. Impact of RM and its components, 1,8-cineole and α-pinene, on *C. albicans***

**RBY1132 intracellular ROS accumulation.**

Merged (bright-field/fluorescence) (left) and fluorescence (right) images show a strong fluorescent DDF signal in *C. albicans* RBY1132 treated with RM, 1,8-cineole and α-pinene at MIC and 1/2 MIC, absent at lower MICs and controls. Scale bars for representative control are 5 μm and applicable to all.
