# Supplementary material for: Rosemary essential oil and its components 1,8-cineole and α-pinene induce ROS-dependent lethality and ROS-independent virulence inhibition in Candida albicans
Source: PLoS One. 2022 Nov 16;17(11):e0277097. doi: 10.1371/journal.pone.0277097 (PMC9668159; doi:10.1371/journal.pone.0277097)
Supplement: S6 Fig — (DOCX) [file pone.0277097.s006.docx]

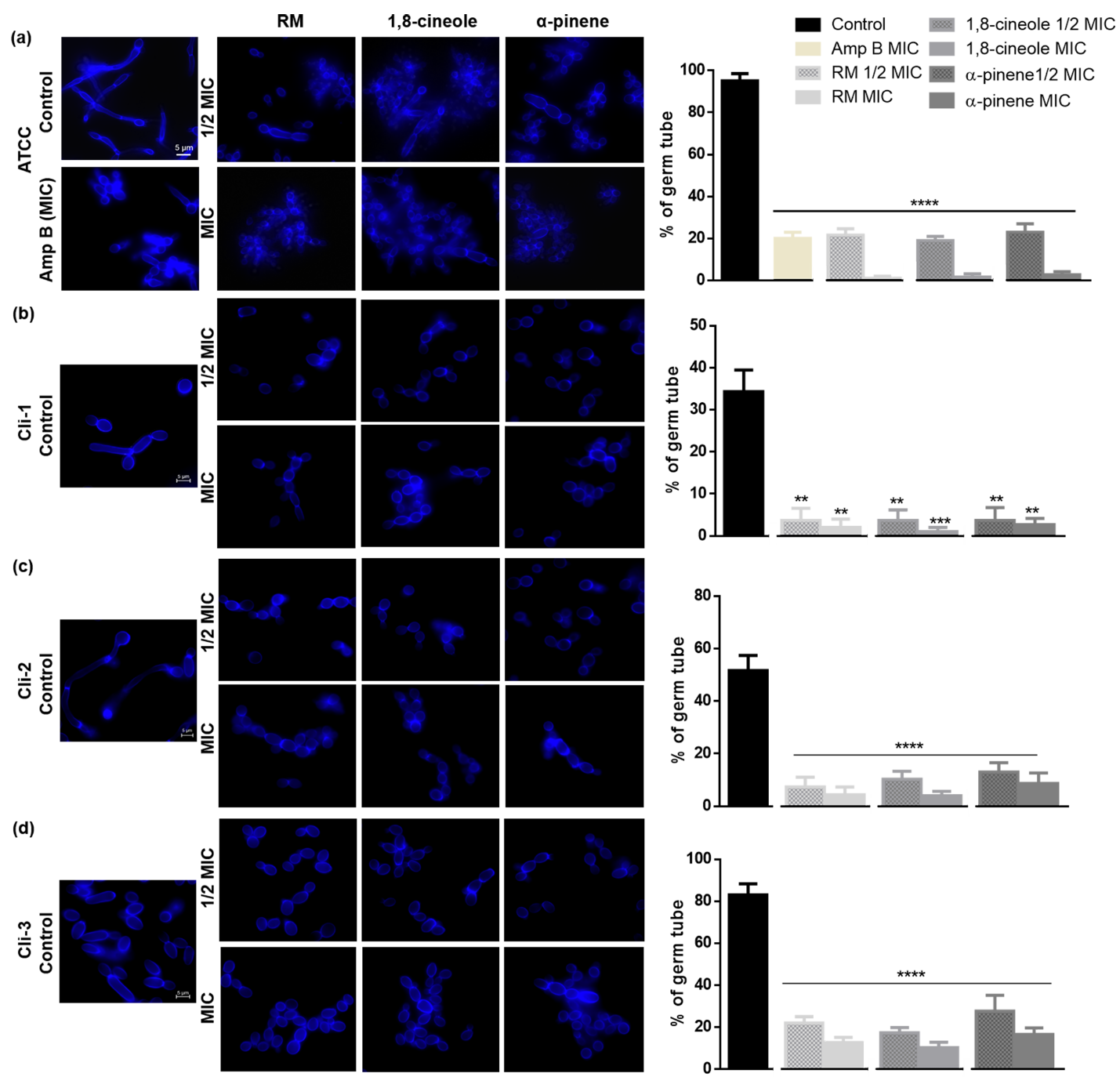


**S6 Fig. Effects of RM, 1,8-cineole and α-pinene on germ tube formation by *C.***

***albicans* ATCC10231 and clinical strains (Cli-1, Cli-2 and Cli-3).**

(a-d, left) Epiflourescence images (λ_ex_ = 365 nm; λ_em_ = 435 nm) show pseudohyphae, aggregates and some regenerated germ tubes in response to EO(C) exposure at 1/2 MIC and MIC. Scale bars for control are 5 μm and applicable for all. (a-d, right) Bar graphs quantify percent germ tube formation, presented as the mean ± SEM of three biological replicates, with 300 cells per replicate, for which statistical significance (****, *p* < 0.0001, ***, *p* < 0.001, **, *p* < 0.01, *, *p* < 0.05) was analysed by an unpaired Student’s *t*-test. All three compounds significantly inhibited the transition of *C. albicans* strains from yeast to hyphae.
