# Supplementary material for: Rosemary essential oil and its components 1,8-cineole and α-pinene induce ROS-dependent lethality and ROS-independent virulence inhibition in Candida albicans
Source: PLoS One. 2022 Nov 16;17(11):e0277097. doi: 10.1371/journal.pone.0277097 (PMC9668159; doi:10.1371/journal.pone.0277097)
Supplement: S7 Fig — (DOCX) [file pone.0277097.s007.docx]

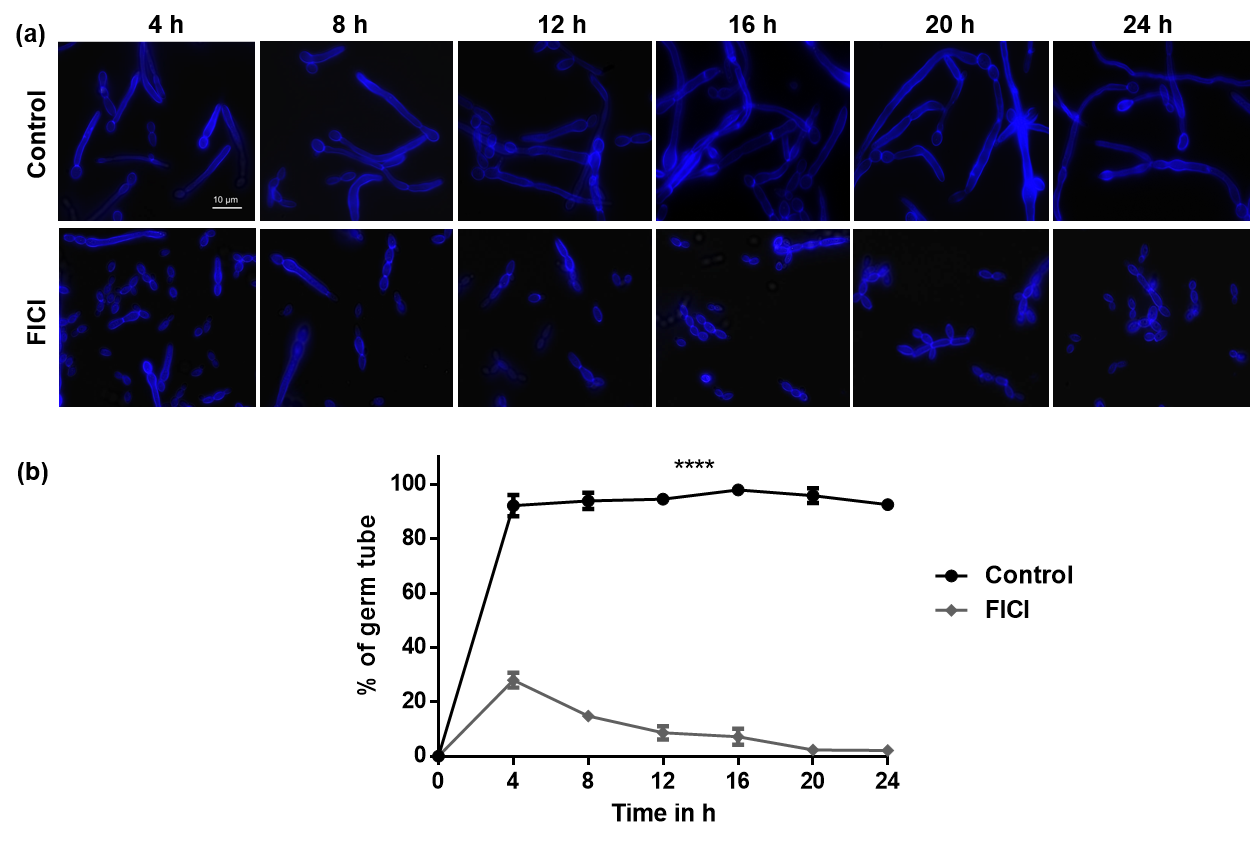


**S7 Fig. Kinetics of *C. albicans* RSY150 germ tube inhibition by 1,8-cineole and α-**

**pinene at FICI**.

(a) Representative epifluorescence (λ_ex_ = 365 nm; λ_em_ = 435 nm) images show the presence or absence of germ tubes following 4, 8, 12, 16, 20 and 24 h exposure to oils at FICI (1/2 MIC 1,8-cineole + 1/8 MIC α-pinene). Scale bar is 10 μm, applicable to all images. (b) Quantification of the combined impact of these two components at FICI on *C.* *albicans* (300 cells/time point) show a significant decrease (****, *p* < 0.0001) in germ tube formation as a function of exposure time, as evaluated by a one-way ANOVA test with Dunnett's multiple comparison.
