# Supplementary material for: Rosemary essential oil and its components 1,8-cineole and α-pinene induce ROS-dependent lethality and ROS-independent virulence inhibition in Candida albicans
Source: PLoS One. 2022 Nov 16;17(11):e0277097. doi: 10.1371/journal.pone.0277097 (PMC9668159; doi:10.1371/journal.pone.0277097)
Supplement: S8 Fig — (DOCX) [file pone.0277097.s008.docx]

**
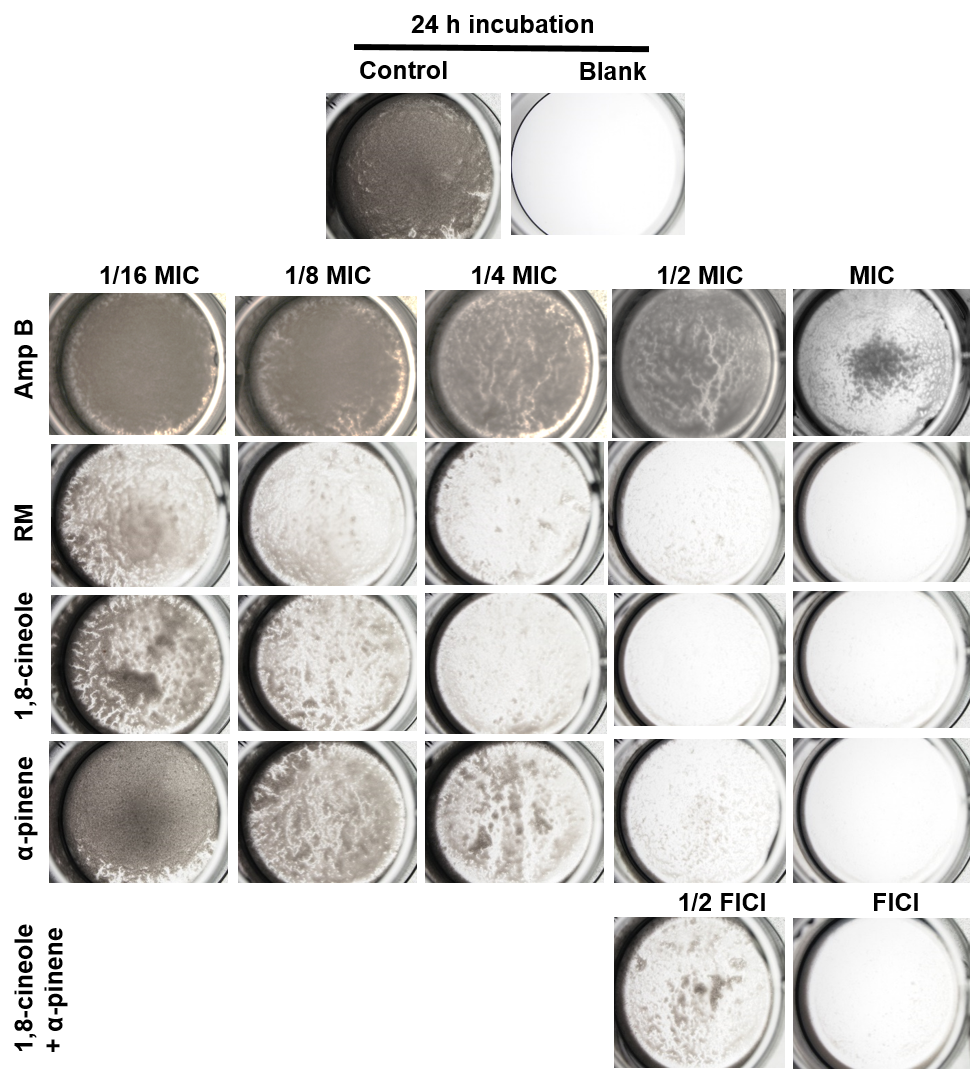
**

**S8 Fig.** **RM oil and its major components reduced biofilm formation in *C. albicans***

**RSY150.**

Stereoscopic bright field images show the visual difference between biofilm mass for *C. albicans* exposed to RM, 1,8-cineole, and α-pinene (from 1/16 MIC to MIC), and the two components at 1/2 FICI and FICI as compared to controls.
