# Supplementary material for: Rosemary essential oil and its components 1,8-cineole and α-pinene induce ROS-dependent lethality and ROS-independent virulence inhibition in Candida albicans
Source: PLoS One. 2022 Nov 16;17(11):e0277097. doi: 10.1371/journal.pone.0277097 (PMC9668159; doi:10.1371/journal.pone.0277097)
Supplement: S9 Fig — (DOCX) [file pone.0277097.s009.docx]

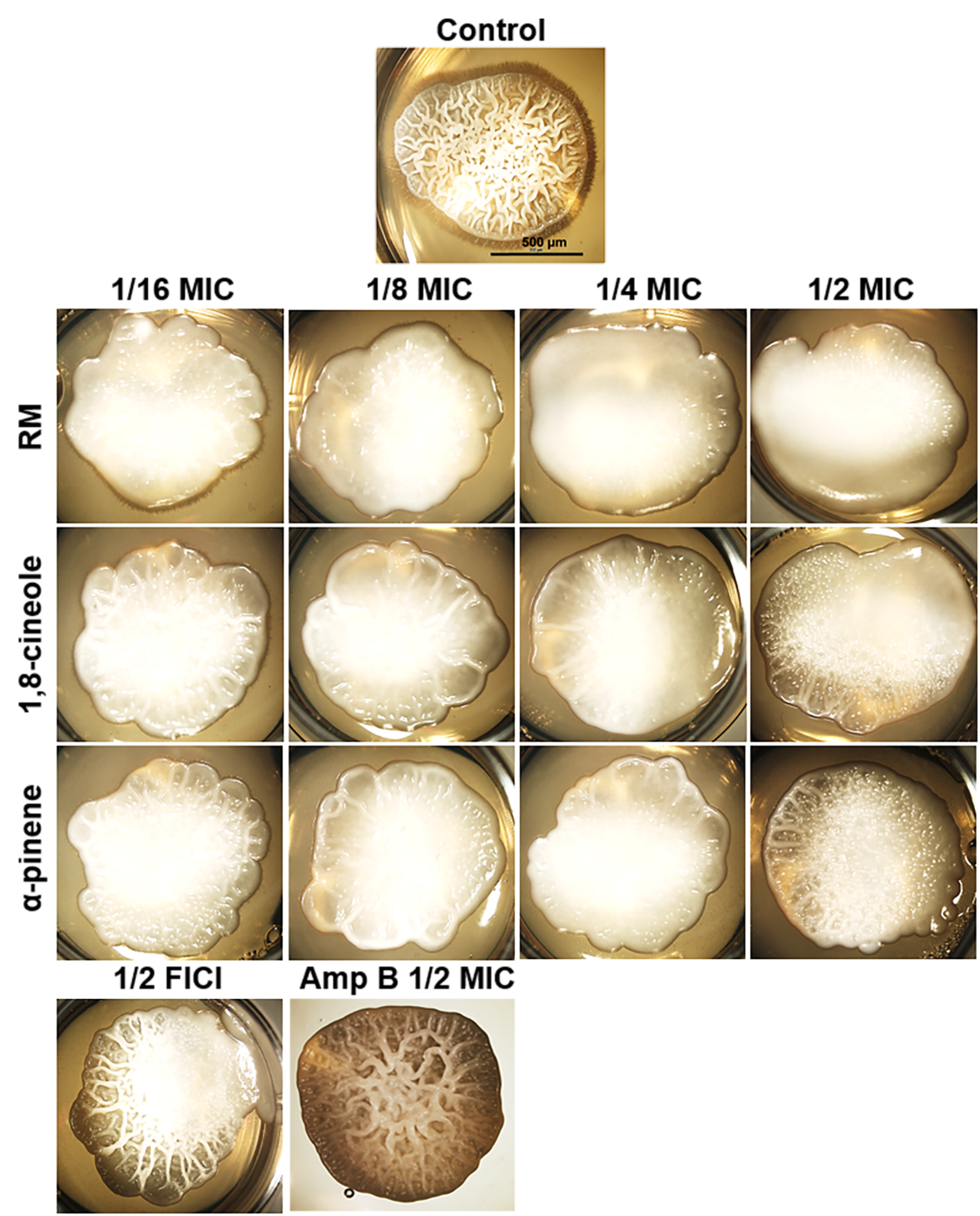


**S9 Fig. Impact of RM oil, 1,8-cineole, and α-pinene on *C. albicans* RSY150 mycelium formation on spider media agar plates (whole colony).**

Representative images show reduced mycelia and wrinkled colonies formed by *C. albicans* RSY 150 when treated with EO(C)s as compared to control. Images were recorded with a digital camera on a bright-field stereo microscope. Bar is 500 µm, applicable to all images.
