# Supplementary material for: Rosemary essential oil and its components 1,8-cineole and α-pinene induce ROS-dependent lethality and ROS-independent virulence inhibition in Candida albicans
Source: PLoS One. 2022 Nov 16;17(11):e0277097. doi: 10.1371/journal.pone.0277097 (PMC9668159; doi:10.1371/journal.pone.0277097)
Supplement: S10 Fig — (DOCX) [file pone.0277097.s010.docx]

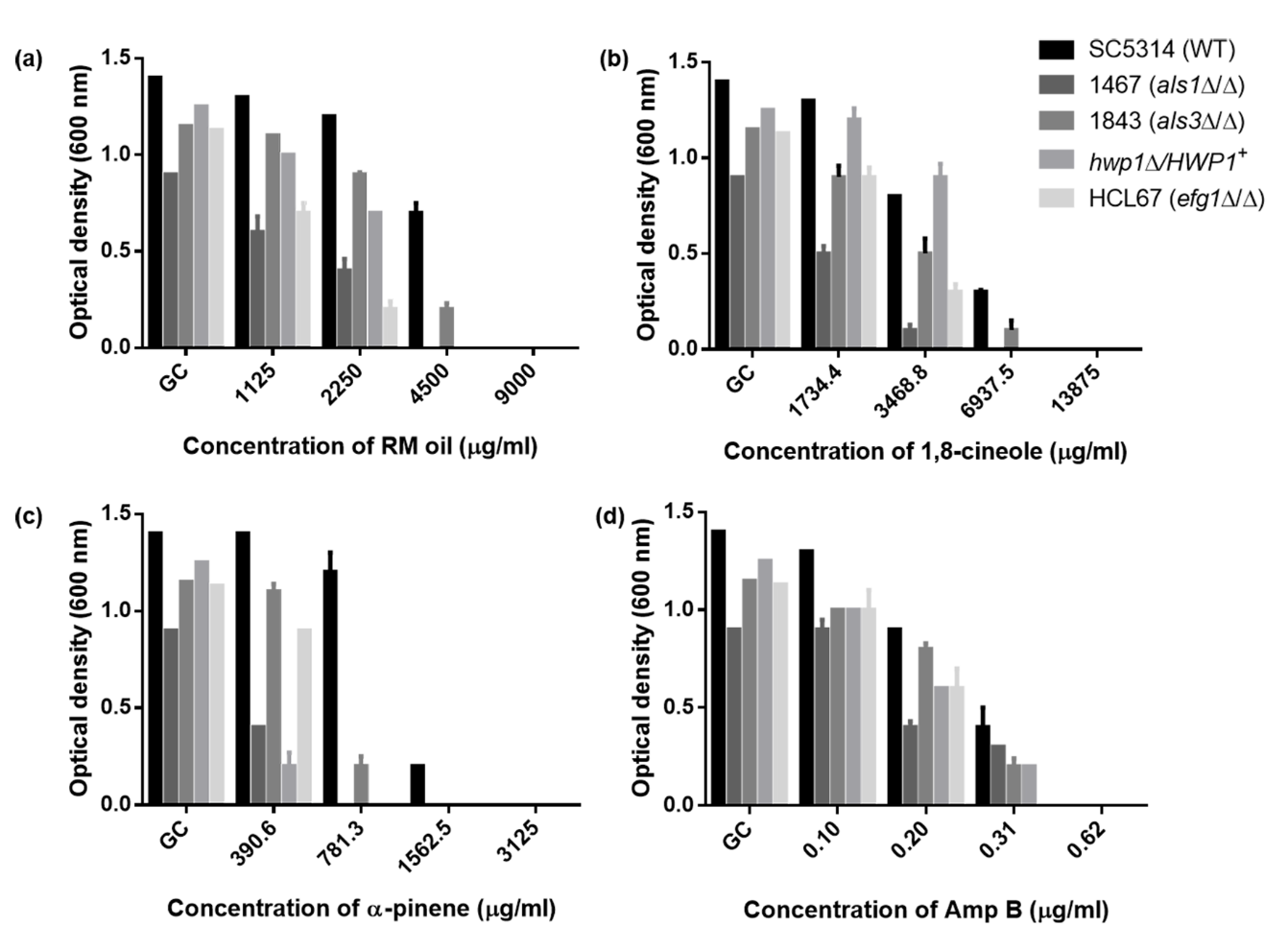


**S10 Fig. Sensitivity profile of *C. albicans* *als1*Δ/Δ, *als3*Δ/Δ*, hwp1*Δ/*HWP1*^+^** **and *efg1*Δ/Δ to EO(C)s.**

(a‒d) Strains were exposed to a two-fold increasing dilution of EO(C)s and evaluated by MIC assay, showing overall sensitivity to RM oil, 1,8-cineole, α-pinene and Amp B. GC = growth control for the background (SC5314) and mutant strains.
