# Supplementary material for: Rosemary essential oil and its components 1,8-cineole and α-pinene induce ROS-dependent lethality and ROS-independent virulence inhibition in Candida albicans
Source: PLoS One. 2022 Nov 16;17(11):e0277097. doi: 10.1371/journal.pone.0277097 (PMC9668159; doi:10.1371/journal.pone.0277097)
Supplement: S11 Fig — (DOCX) [file pone.0277097.s011.docx]

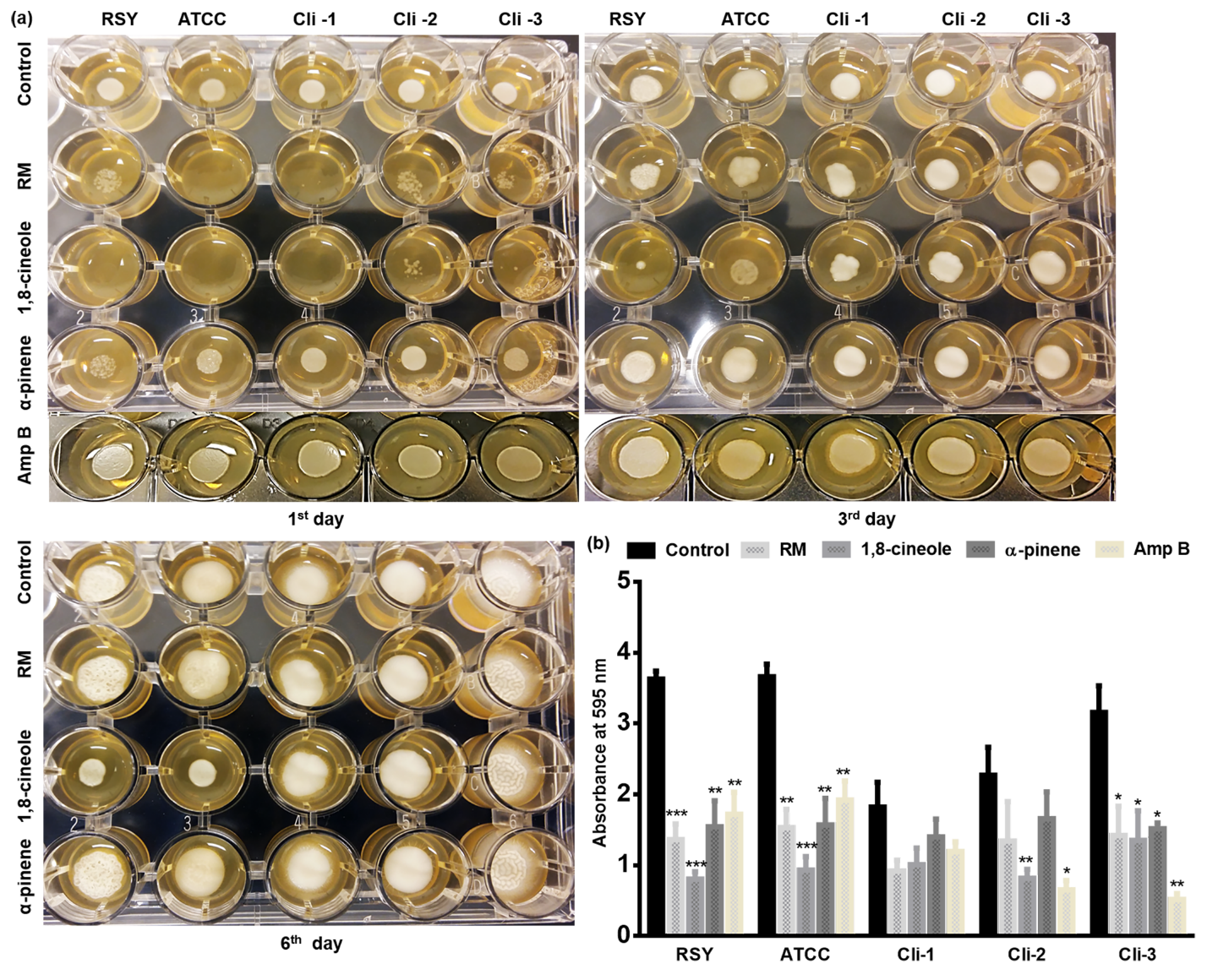


**S11 Fig.** **Impact of pre-treating *C. albicans* RSY150, ATCC10231 and clinical isolates (Cli-1, 2 and 3) with RM, 1,8-cineole and α-pinene at 1/2 MIC on hyphal and biofilm formation.**

(a) One day incubation showed slow growth as compared to control, whereas subsequent incubation to day 3 showed colony formation. At the end of day 6, treated cells were able to produce mycelial growth, except in the presence of 1,8-cineole which had more prominent effects on RSY150, and ATCC10231 was unable to produce any mycelia. (b) Biofilm formation by *C. albicans* strains after pre-treatment with RM, 1,8-cineole, and α-pinene at ½ MIC evaluated by the colorimetric crystal violet assay shows reduced *Candida* biofilm formation with the exception of Cli-1, a genital strain. Data are presented as the mean ± SEM of four biological replicates for which statistical significance (***, *p* < 0.001; **, *p* < 0.01; *, *p* < 0.05) was analysed by a one-way ANOVA, followed by Dunnett’s multiple comparison of each condition versus control.
