# Supplementary material for: Rosemary essential oil and its components 1,8-cineole and α-pinene induce ROS-dependent lethality and ROS-independent virulence inhibition in Candida albicans
Source: PLoS One. 2022 Nov 16;17(11):e0277097. doi: 10.1371/journal.pone.0277097 (PMC9668159; doi:10.1371/journal.pone.0277097)
Supplement: S1 Table — (DOCX) [file pone.0277097.s012.docx]

**S1 Table.** *C. albicans* strains used in this study.

| **Strains** | **Genotype/ Isolate description** | **References** |
| --- | --- | --- |
| RBY1132^a^ | *his1^−^/ ^−^ leu2^−^/ ^−^ arg4^−^/ ^−^* | [1] |
| RSY150^a^ | *TUB2-GFP-SAT1/TUB2^+^ HTB1-RFP-ARG4^+^/HTB1^+^ arg4^−^/ ^−^* | [1] |
| ATCC 10231 | Clinical reference strain | RQHR^b^,  Regina  SK, Canada |
| SC5314 | Clinical isolate – wild type | [2] |
| *C. albicans*  (1 ‒ 3) | Clinical isolates; 2 genital and 1 blood strains | RQHR, Regina, SK, Canada |
|  | ***C. albicans* knockout mutants** |  |
| **Knockout mutant** | **Function/ Genotype description** |  |
| *C. albicans* | GRACE 1.0 library | [3] |
| *hwp1*Δ/HWP1*^+^* | *his3::hisG/his3::hisGleu2::tetRGAL4AD-URA3/LEU2-HWP1* | [3] |
| 1467 (*als1*Δ/Δ) | *iro1-ura3∆ : : λimm^434^/iro1-ura3∆ : : λimm^434^ als1sa∆/als1la∆-URA3*^c^ | [4] |
| 1843 (*als3*Δ/Δ) | iro1-ura3∆ : : λimm^434^/iro1-ura3∆ : :λ imm^434^ *als3la*∆/*als3sa*∆-URA3^c^ | [4] |
| HLC67 (*efg1*Δ/Δ) | *CAI4 efg1::hisG/efg1::hisG MTL*a/α^d^ | [5] |

^a^The full genotype at the auxotrophic markers for RSY is as follows: *his1::hisG/his1::hisGleu2::hisG/leu2::hisGarg4::hisG/arg4::hisGura3*::*imm434::URA3/ura3::imm434 iro1::IRO1/iro1::imm434*, as described by Sherwood and Bennett [1].

^b^RQHR - Regina Qu'Appelle Health Region

^c^Alleles of ALS genes are marked with LA to designate the large allele or SA for the small allele in strain SC5314.

^d^*MTL -* Mating type like

1. Sherwood RK, Bennett RJ. Microtubule motor protein Kar3 is required for normal mitotic division and morphogenesis in Candida albicans. Eukaryotic cell. 2008;7(9):1460-74. Epub 2008/07/01. doi: 10.1128/ec.00138-08. PubMed PMID: 18586948; PubMed Central PMCID: PMCPMC2547067.

2. Gillum AM, Tsay EY, Kirsch DR. Isolation of the Candida albicans gene for orotidine-5'-phosphate decarboxylase by complementation of S. cerevisiae ura3 and E. coli pyrF mutations. Molecular & general genetics : MGG. 1984;198(2):179-82. Epub 1984/01/01. doi: 10.1007/bf00328721. PubMed PMID: 6394964.

3. Roemer T, Jiang B, Davison J, Ketela T, Veillette K, Breton A, et al. Large-scale essential gene identification in Candida albicans and applications to antifungal drug discovery. Molecular microbiology. 2003;50(1):167-81. Epub 2003/09/26. doi: 10.1046/j.1365-2958.2003.03697.x. PubMed PMID: 14507372.

4. Zhao X, Oh SH, Cheng G, Green CB, Nuessen JA, Yeater K, et al. ALS3 and ALS8 represent a single locus that encodes a Candida albicans adhesin; functional comparisons between Als3p and Als1p. Microbiology (Reading, England). 2004;150(Pt 7):2415-28. Epub 2004/07/17. doi: 10.1099/mic.0.26943-0. PubMed PMID: 15256583.

5. Lo HJ, Köhler JR, DiDomenico B, Loebenberg D, Cacciapuoti A, Fink GR. Nonfilamentous C. albicans mutants are avirulent. Cell. 1997;90(5):939-49. Epub 1997/09/23. doi: 10.1016/s0092-8674(00)80358-x. PubMed PMID: 9298905.
