# Supplementary material for: Rosemary essential oil and its components 1,8-cineole and α-pinene induce ROS-dependent lethality and ROS-independent virulence inhibition in Candida albicans
Source: PLoS One. 2022 Nov 16;17(11):e0277097. doi: 10.1371/journal.pone.0277097 (PMC9668159; doi:10.1371/journal.pone.0277097)
Supplement: S2 Table — (DOCX) [file pone.0277097.s013.docx]

**S2 Table.** Concentrations of essential oil stock and working solutions used in this study.

| **Name of oils / Components** | **Stock solutions**  **(µg /mL)** | **Working concentrations (µg/mL)** |
| --- | --- | --- |
| RM | 36, 000 | 8.9 – 18000 |
| 1,8-cineole | 37, 000 | 9.0 – 18500 |
| α-pinene | 12, 500 | 3.0 – 6250 |
