# Supplementary material for: Rosemary essential oil and its components 1,8-cineole and α-pinene induce ROS-dependent lethality and ROS-independent virulence inhibition in Candida albicans
Source: PLoS One. 2022 Nov 16;17(11):e0277097. doi: 10.1371/journal.pone.0277097 (PMC9668159; doi:10.1371/journal.pone.0277097)
Supplement: S3 Table — (DOCX) [file pone.0277097.s014.docx]

**S3 Table.** Composition of rosemary (*Rosmarinus officinalis*) essential oil from whole plants analyzed by GC/MS.

| **Compound** | **% of Yields** | **Experimental RI^a^** | **Literature Reference RI^b^** |
| --- | --- | --- | --- |
| α-thujene | 0.2 | 928 | 931 |
| **α-pinene** | **12** | **936** | **941** |
| camphene | 4.0 | 950 | 953 |
| β-pinene | 7.3 | 979 | 979 |
| Myrcene | 0.8 | 992 | 988 |
| α-phellandrene | 0.2 | 1006 | 1007 |
| 3-carene | 0.2 | 1012 | 1010 |
| α-terpinene | 0.2 | 1019 | 1016 |
| *p*-cymene | 1.8 | 1027 | 1021 |
| **1,8-cineole** | **53*** | **1035** | **1033** |
| γ-terpinene | 0.7 | 1060 | 1057 |
| terpinolene | 0.3 | 1090 | 1080 |
| Linalool | 0.6 | 1101 | 1094 |
| Camphor | 8.6 | 1149 | 1136 |
| Borneol | 0.9 | 1170 | 1162 |
| terpin-4-ol | 0.9 | 1180 | 1179 |
| α-terpineol | 1.7 | 1193 | 1184 |
| bornyl acetate | 0.7 | 1290 | 1281 |
| β-caryophyllene | 4.4 | 1426 | 1422 |
| α-humulene | 0.3 | 1460 | 1443 |
| **Total** | **98.8** |  |  |

^a^RI = retention index ; Estimated error: ± 0.1 % (area) and ± 1 for retention

indices between trials; Retention index on a HP-5 column with reference to

*n*-alkanes [1].

^b^MS = mass spectrometric analysis; The relative area under the peak was

calculated and experimental data compared with mass spectral NIST library

spectra and the literature [2].

1. Joulain D, König WA. The Atlas of Spectral Data of Sesquiterpene Hydrocarbons: E.B.-Verlag; 1998.
2. Adams RP. Identification of essential oil components by gas chromatography/mass spectrometry: Allured publishing corporation Carol Stream, IL; 2007.
