# Supplementary material for: Rosemary essential oil and its components 1,8-cineole and α-pinene induce ROS-dependent lethality and ROS-independent virulence inhibition in Candida albicans
Source: PLoS One. 2022 Nov 16;17(11):e0277097. doi: 10.1371/journal.pone.0277097 (PMC9668159; doi:10.1371/journal.pone.0277097)
Supplement: S4 Table — (DOCX) [file pone.0277097.s015.docx]

**S4 Table.** CDC gene ontology functional analysis of *C. albicans* genes conferring significant EO(C)-resistance and -sensitivity.

| **Terms from the Function Ontology** | **Resistant genes** | | **Sensitive genes** | |
| --- | --- | --- | --- | --- |
|  | **1,8-cineole** | **α-pinene** | **1,8-cineole** | **α-pinene** |
| **Gene Ontology term** | Quinone binding | Carboxypeptidase activity | None | Alpha-1,2-mannosyltransferase activity |
| **Cluster frequency** | 2 out of 40 genes, 5.0% | 2 out of 50 genes, 4.0% |  | 2 out of 36 genes, 5.6% |
| **Background frequency** | 6 out of 6473 background genes, 0.1% | 6 out of 6473 background genes, 0.1% |  | 9 out of 6473 background genes, 0.1% |
| **Corrected P-value** | 0.02749 | 0.04558 |  | 0.03803 |
| **False discovery rate** | 14.00% | 70.00% |  | 54.00% |
| **Genes annotated to the term** | AMO1 (Un- characterized, putative peroxisomal copper amine oxidase), FESUR1 (Verified, Putative ubiquinone reductase) | CPY1 (Un- characterized, Carboxypeptidase Y), ECM14 (Verified, putative metallo-carboxypeptidase) |  | C5_05040W_A (Un-characterized, Putative mannosyltransferase), MNT2 (Verified, lpha-1,2-mannosyl transferase) |
