# Supplementary material for: Rosemary essential oil and its components 1,8-cineole and α-pinene induce ROS-dependent lethality and ROS-independent virulence inhibition in Candida albicans
Source: PLoS One. 2022 Nov 16;17(11):e0277097. doi: 10.1371/journal.pone.0277097 (PMC9668159; doi:10.1371/journal.pone.0277097)
Supplement: S5 Table — (DOCX) [file pone.0277097.s016.docx]

**S5 Table.** Relative germ tube inhibition by RM oil and its components at MIC.

| **Strains** | ***Significance***  ***RM and α-pinene*** | ***Significance***  ***1,8 cineole*** |
| --- | --- | --- |
| **RSY 150** | *p* < 0.0001 | *p* < 0.0001 |
| **ATCC10231** | *p* < 0.0001 | *p* < 0.0001 |
| **Cli-1 (genital)** | *p* < 0.01 | *p* < 0.001 |
| **Cli-2 (genital)** | *p* < 0.001 | *p* < 0.001 |
| **Cli-3 (blood)** | *p* < 0.001 | *p* < 0.001 |
