# Supplementary material for: Rosemary essential oil and its components 1,8-cineole and α-pinene induce ROS-dependent lethality and ROS-independent virulence inhibition in Candida albicans
Source: PLoS One. 2022 Nov 16;17(11):e0277097. doi: 10.1371/journal.pone.0277097 (PMC9668159; doi:10.1371/journal.pone.0277097)
Supplement: S6 Table — (DOCX) [file pone.0277097.s017.docx]

**S6 Table.** Relative reduction in biofilm formation following pre-treatment with RM oil and its components at 1/2 MIC.

| **Strains** | **Significance**  **RM** | **Significance**  **1,8 cineole** | **Significance**  **α-pinene** | **Significance**  **Amp B** |
| --- | --- | --- | --- | --- |
| **RSY 150** | *p* < 0.001 | *p* < 0.001 | *p* < 0.01 | *p* < 0.05 |
| **ATCC10231** | *p* < 0.01 | *p* < 0.001 | *p* < 0.01 | *p* < 0.01 |
| **Cli-1 (genital)** | NS* | NS | NS | NS |
| **Cli-2 (genital)** | NS | *p* < 0.01 | NS | *p* < 0.05 |
| **Cli 3 (blood)** | *p* < 0.05 | *p* < 0.05 | *p* < 0.05 | *p* < 0.01 |

*NS- not significant
